# Impact of long-term dietary habits on the human gut resistome in the Dutch population

**DOI:** 10.1038/s41598-022-05817-4

**Published:** 2022-02-03

**Authors:** Paul B. Stege, Joost Hordijk, Sudarshan A. Shetty, Michael Visser, Marco C. Viveen, Malbert R. C. Rogers, Esther Gijsbers, Cindy M. Dierikx, Rozemarijn Q. J. van der Plaats, Engeline van Duijkeren, Eelco Franz, Rob J. L. Willems, Susana Fuentes, Fernanda L. Paganelli

**Affiliations:** 1 Department of Medical Microbiology, University Medical Center Utrecht, Utrecht University, Heidelberglaan 100, 3584 CX Utrecht, The Netherlands; 2 Centre for Infectious Disease Control Netherlands, National Institute for Public Health and the Environment (RIVM), Bilthoven, The Netherlands; 3 Present Address: National Plant Protection Organization (NPPO-NL), The Netherlands Food and Consumer Product Safety Authority (NVWA), 6700 HC Wageningen, The Netherlands

**Keywords:** Molecular biology, Antimicrobials, Antibiotics, Microbiology, Microbial communities, Metagenomics, Microbiome

## Abstract

The human gut microbiome plays a central role in health and disease. Environmental factors, such as lifestyle and diet, are known to shape the gut microbiome as well as the reservoir of resistance genes that these microbes harbour; the resistome. In this study we assessed whether long-term dietary habits within a single geographical region (the Netherlands) impact the human gut resistome. Faecal samples from Dutch omnivores, pescatarians, vegetarians and vegans were analysed by metagenomic shotgun sequencing (MSS) (n = 149) and resistome capture sequencing approach (ResCap) (n = 64). Among all diet groups, 119 and 145 unique antibiotic resistance genes (ARGs) were detected by MSS or ResCap, respectively. Five or fifteen ARGs were shared between all diet groups, based on MSS and ResCap, respectively. The total number of detected ARGs by MSS or ResCap was not significantly different between the groups. MSS also revealed that vegans have a distinct microbiome composition, compared to other diet groups. Vegans had a lower abundance of *Streptococcus thermophilus* and *Lactococcus lactis* compared to pescatarians and a lower abundance of *S. thermophilu*s when compared to omnivores. In summary, our study showed that long-term dietary habits are not associated with a specific resistome signature.

## Introduction

The human gut microbiome is a complex ecosystem composed of bacteria, fungi, viruses and phages. It not only plays a central role in nutrient acquisition, but it also affects our state of health and disease^1–3^. Many factors are known to influence its composition, which can be either host-derived, such as age or immunological and pathological disorders^4,5^, or exposure to environmental factors (the exposome), including diet^6–12^. Correlations have been observed between diets rich in protein and animal-fats and the high relative abundance of *Bacteroides*, as opposed to carbohydrate-rich diets and the high abundance of *Prevotella*^13,14^. In addition, increased abundance of *Prevotella* and *Lachnospira* was correlated with fiber- and vegetable-rich diets^15,16^. The majority of studies that have observed these diet-induced effects on the microbiome either compare participants from different geographic areas or involve short term dietary intervention studies^14,17–19^. When studying the effect of long-term dietary habits within a single community on the gut microbiome of vegans, vegetarians and omnivores, Losasso et al. only observed differences in bacteria that are present in low abundance, and part of the families *Bacteroides*, *Lachnospiraceae*, and *Ruminococcaceae*^20^. This study used 16S rRNA gene sequencing to determine the microbiome composition, which has insufficient resolution to allow for comparisons at the species level.

The human gut microbiome is also an important reservoir of antibiotic resistance genes (ARGs)^21–24^. It is therefore important to understand how long-term dietary habits not only impact the microbiome composition, but also the composition of the total of ARGs, the resistome, in the human gut. Advances in high-throughput sequencing have allowed in depth studies of the human gut resistome. The gut resistome of healthy humans can typically contain over 100 unique ARGs, with the most abundant ARGs being those that encode for tetracycline resistance, followed by macrolide and beta lactam resistance genes^25,26^. Just as with the microbiome, several factors are known to alter the resistome composition. Orally administered antibiotics are known to select for bacteria that are resistant to these antibiotics, therefore increasing the abundance of these ARGs in the gut^27,28^. This antibiotic-driven enrichment can take place in either the general population or more specific, in clinical settings, where effects on the resistome were observed in a matter of days^29–31^. Additionally, the human gut resistome can be affected by environmental factors such as international travel and living conditions. This has been shown in studies comparing urbanized with agricultural populations, in which the use of antibiotics plays an important role^32,33^. Finally, meat contaminated with bacteria carrying ARGs as a result of antibiotic usage in livestock, has been highlighted as a possible transmission route for resistant bacteria and could therefore influence the gut resistome as well^34–38^. More specifically, zoonotic pathogens such as species of *Salmonella* and *Campylobacter* and certain types of *Escherichia coli* (*e.g.* Shiga-toxin-producing *E. coli* (STEC)) are known for causing foodborne infections and are frequent carriers of ARGs^39–42^.

In this study we assessed whether long-term dietary habits within a single geographical region impact the human gut resistome in the general Dutch population. Using metagenomic shotgun sequencing (MSS), we were able to detect 877 unique bacterial species and an extensive resistome composed of 119 unique ARGs, in the gut microbiome of healthy Dutch residents. resistome capture sequencing approach (ResCap) was applied for a subset of samples and revealed 145 unique ARGs, thereby surpassing the detection limits of MSS. Despite the high resolution of the sequencing data, the total number of ARGs detected by MSS or ResCap per diet group was not significantly different in the general Dutch population.

## Results

### Diet-associated differences in the gut microbiome

**Figure 1:**
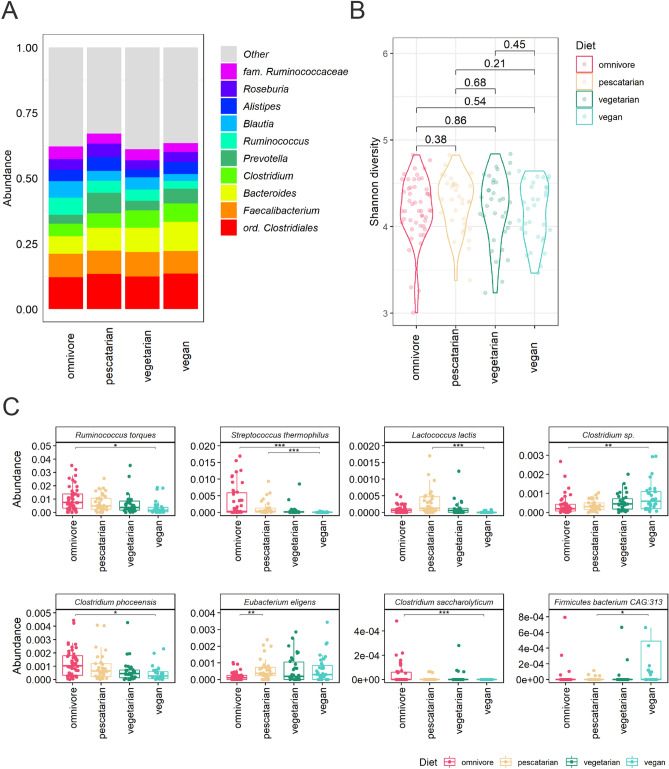
Association of dietary habits and the gut microbiome composition. (**A**) Relative abundance of the 10 most abundant bacterial genera per diet group. (**B**) Alpha diversity per diet group expressed by Shannon index. Differences in alpha diversity between diet groups were compared using Wilcoxon rank-sum tests. (**C**) ANCOM-BC analysis for differential relative abundance of bacterial species between diet groups. Abundance was plotted on the relative abundance scale from 0.00 to 1.00. Adjusted p values are indicated by *< 0.05, **< 0.01 and ***< 0.001.

Before determining diet-associated resistome differences, we first assessed the effect of long-term dietary habits on the gut microbiome, as this ecological niche is an important reservoir of antibiotic resistance genes. Faecal samples from 149 Dutch individuals were selected based on their dietary habits and categorized in four matched diet groups: (1) omnivores, (2) pescatarians, (3) vegetarians and (4) vegans (Table 1). Faecal samples from these four groups were used for metagenomic shotgun sequencing (MSS) in order to study the effect of dietary habits on the gut microbiome. The mOTUs2-based taxonomic binning method revealed that the top 10 most abundant genera did not differ between the diet groups (Fig. 1a). In all our study groups, the most abundant taxa belong to the order of *Clostridiales* and genera *Faecalibacterium*, *Bacteroides*, *Clostridium* and *Prevotella*, which matches with the gut microbiome composition observed in previous studies^43,44^. In addition, the gut microbiome diversity, expressed as Shannon index, was not significantly different between diet groups, indicating that the total species diversity is highly similar among diet groups (Fig. 1b). Statistical analysis of the inter-diet group beta diversity based principal component analysis (PCA) using Aitchison distance, Bray–Curtis distance, or Jaccard distance further revealed that diet was not a main driver of the observed variance in microbiome composition between the samples (figure s1).

**Table 1:** Study participants characteristics included in metagenomic shotgun sequencing.

Characteristics	Omnivore	Pescatarian	Vegetarian	Vegan
Participants, n = 149	50	33	34	32
Age in years, *median *(10th–90th percentile)	47 (29–59)	51 (29–62)	45 (28–62)	37 (29–56)
Male participants*,*(percentage)	16 (32%)	12 (36%)	11 (32%)	11 (34%)
Participants with pets, (percentage)	28 (56%)	16 (48%)	18 (53%)	18 (56%)
Participants using medication*, (percentage)	15 (30%)	15 (45%)	7 (21%)	8 (25%)

*Medication other than antibiotics, proton pump inhibitors, insulin and cancer treatment.

We further explored potential differences in the gut microbiome composition between diet groups by using supervised analysis to compare the abundance of bacterial species using ANCOM-BC^45^. Compared to the omnivores, vegans had lower abundance of *Ruminococcus torques* (p_adj_ = 5.0E−02), *Streptococcus thermophilus* (p_adj_ = 4.7E−06), *Clostridium sp.* (p_adj_ = 6.5E−03), *Clostridium phoceensis* (p_adj_ = 3.7E−02) and *Clostridium saccharolyticum* (p_adj_ = 1.6E−03) (Fig. 1c). Similarly, *Streptococcus thermophilus* (p_adj_ = 1.5E−04), *Lactococcus lactis* (p_adj_ = 7.0E−07) and *Firmicutes bacterium* CAG:313 (p_adj_ = 2.7E−02) were less abundant in vegans compared to pescatarians. Finally, *Eubacterium eligens* (p_adj_ = 2.9E−03) was more abundant in the microbiome of pescatarians when compared to omnivores (Fig. 1c).

In addition to mOTUs2 we also applied MetaPhlAn3 to profile the microbiome composition. In concordance with the results of the mOTUs2, using MetaPhlAn3 we did not observe differences in the top 10 most abundant genera between the diet groups (figure s2). Using MetaPhlAn3 for differential abundance analysis, we also found a lower relative abundance of *R. torques*, *S. thermophilus* and *C. saccharolyticum* in vegans compared to omnivores and higher abundance of *E. eligens* in pescatarians compared to omnivores (figure s3). Furthermore, the MetaPhlAn3 approach detected a higher relative abundance of *Lactobacillus delbrueckii*, *Coprococcus comes*, *Dorea formicigenerans*, *Dorea longicatena*, *Lawsonibacter asaccharolyticus* and *Phascolarctobacterium* CAG:266 in omnivores compared to vegans (figure s3).

### Composition of the gut resistome across diet groups based on metagenomic shotgun sequencing

**Figure 2:**
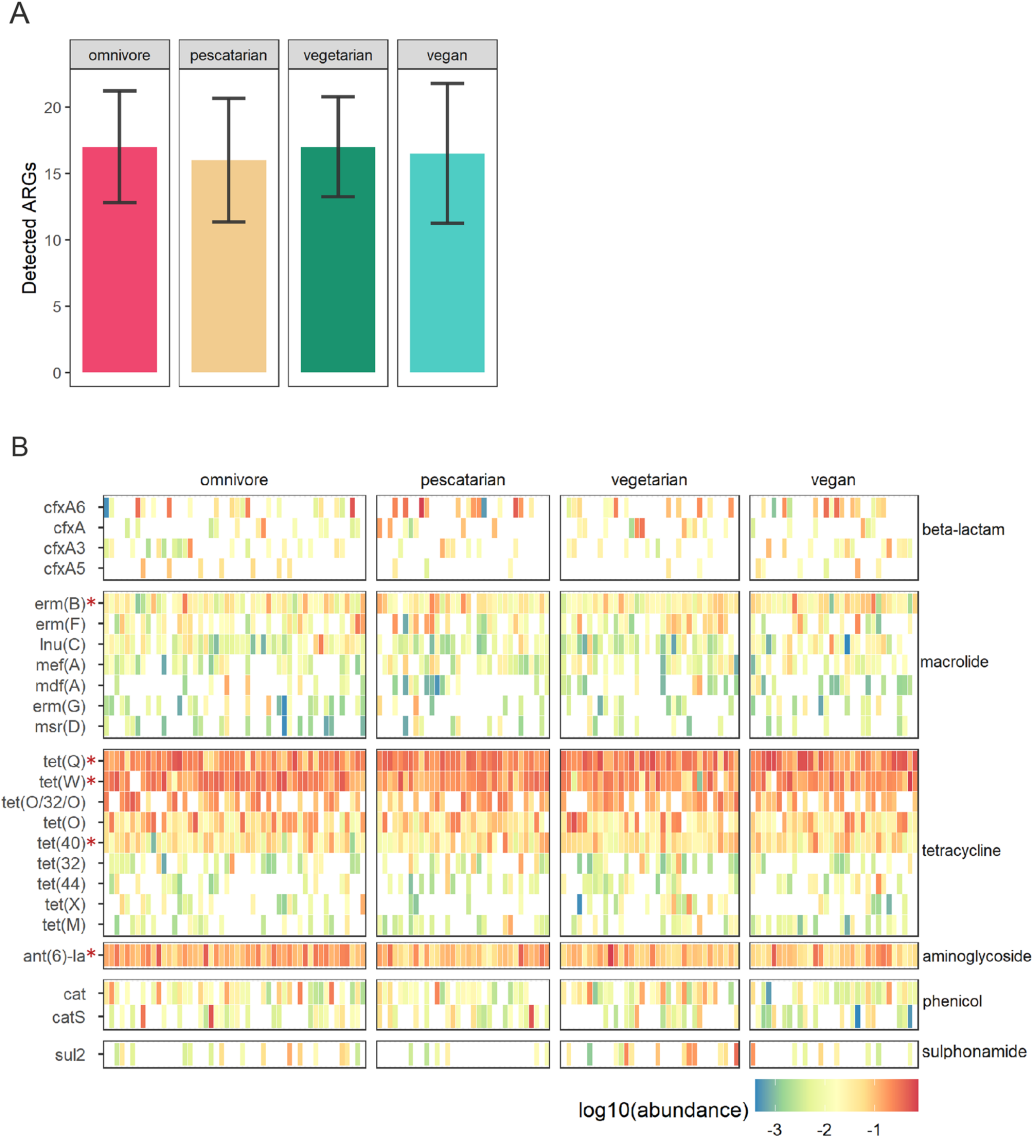
Antibiotic resistance gene distribution in the gut resistome through metagenomic shotgun sequencing. (**A**) Average number and standard deviation of antibiotic resistance genes (ARGs) detected in each diet group. (**B**) Heatmap depicting ARGs abundance per participant. Each column denotes a study participant, clustered per diet group. Rows represent the relative abundance of ARG classes. ARGs present at least in 10% of the participants are shown. ARGs present in the resistome of at least 95% of the participants are indicated by the asterisks.

We next set out to investigate whether long-term dietary habits impacted the gut resistome composition. Using MSS we were able to identify 119 unique antibiotic resistance genes (ARGs) among all diet groups. The total number of detected ARGs was not significantly different between the groups, with an average of 17 ± 4 genes found in omnivores, 16 ± 5 in pescatarians, 17 ± 4 in vegetarians and 17 ± 5 in vegans (Fig. 2a). Among these, five were consistently detected in all diet groups (detected in 95% of the samples), namely the aminoglycoside resistance gene *ant(6)-Ia*, the macrolide-lincosamide-streptogramin B resistance gene *erm*(B), and the tetracycline resistance genes *tet*(40), *tet*(Q) and *tet*(W) (Fig. 2b).

**Figure 3:**
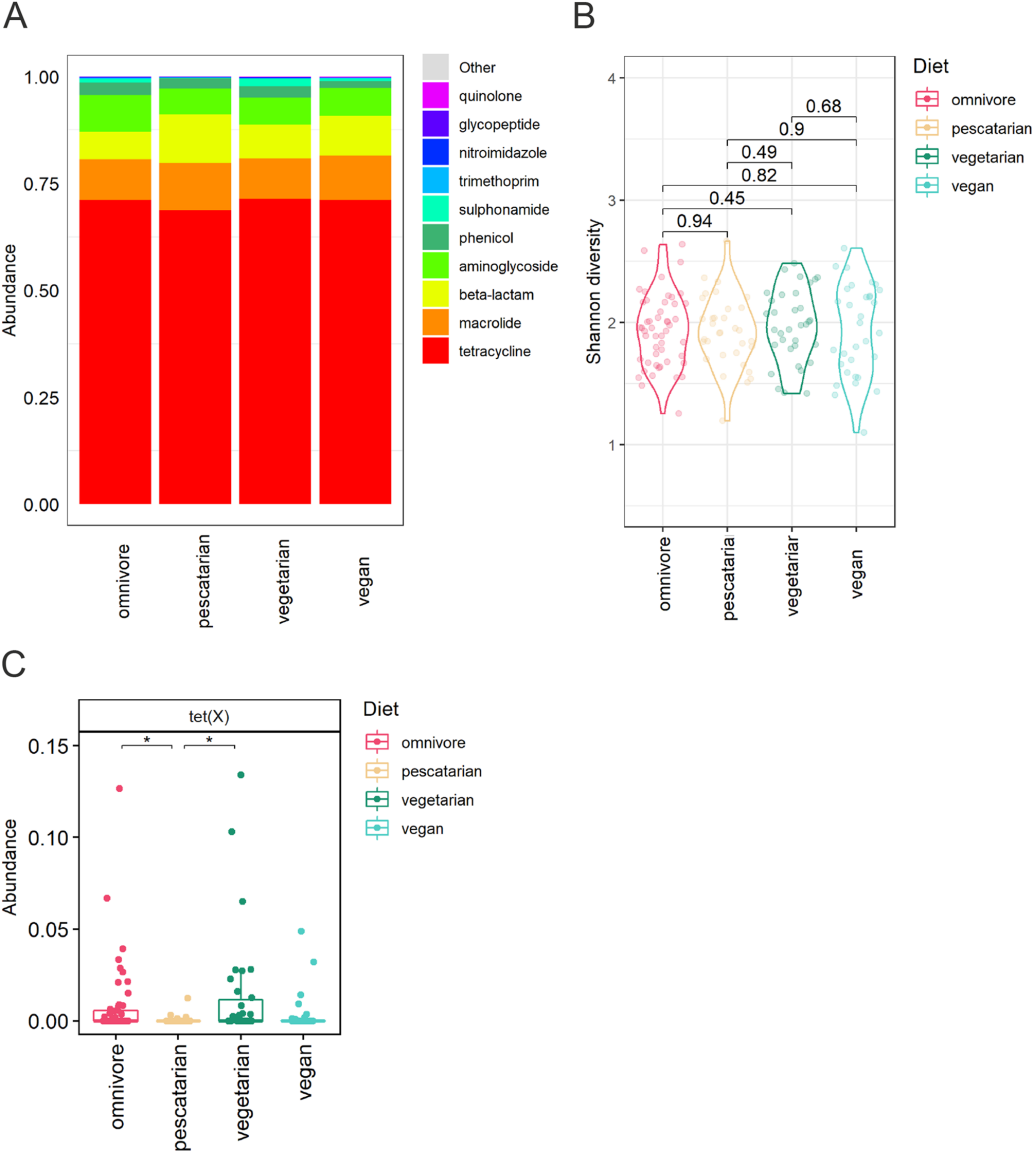
Association of dietary habits and the gut resistome composition through metagenomic shotgun sequencing. (**A**) Relative abundance of the 10 most abundant gene classes encoding antibiotic resistance, summarized per diet group. (**B**) Alpha diversity per diet group expressed by Shannon index. Differences in alpha diversity between diet groups were compared using Wilcoxon rank-sum tests. (**C**) Differential abundance analysis using ANCOM-BC plotted on relative abundance scale from 0.00 to 1.00. Adjusted p values below 0.05 are indicated by *.

The most abundant genes detected encode for resistance to the classes of tetracyclines, macrolides, beta-lactams, aminoglycosides and phenicols. There were no differences when comparing the overall top 10 most abundant ARGs between the diet groups (Fig. 3a). Also, the resistome diversity, expressed by Shannon index, was not significantly different between the diet groups (Fig. 3b). In addition, diet does not seem to be the main driver of the observed variance in resistome composition between the samples when analysed by beta diversity based principal component analysis (PCA, figure s4). Supervised analysis using ANCOM-BC only revealed a significant difference of the abundance of *tet*(X), which is present in low abundance in pescatarians in comparison to omnivores (p_adj_ = 4.4E−02) and vegetarians (p_adj_ = 1.0E−02) (Fig. 3c). Overall, based on MSS, the resistome was highly similar among the studied diet groups.

### Higher antibiotic resistance gene detection sensitivity of ResCap compared to metagenomic shotgun sequencing

Although MSS was able to detect a large variety of ARGs, these only represented 0.06 ± 0.03% of the total number of reads. This indicates that the proportion of ARGs is relatively low when compared to the total gene pool present in the samples. In order to improve the sensitivity to detect ARGs, we applied the probe based resistome capture sequencing approach (ResCap), on a subset (64/149; 43%) of samples (Table 2)^46^. ResCap was able to greatly enrich the number ARGs specific reads, with 40.5 ± 15.2% of the total number of reads sequenced mapping to ARGs.

**Table 2:** Participant characteristics of the subset selected for ResCap.

Characteristics	Omnivore	Pescatarian	Vegetarian	Vegan
Participants, n = 64	16	16	16	16
Age in years, *median *(10th–90th percentile)	46 (27–57)	42 (31–59)	44 (30–59)	43 (33–56)
Male participants, (percentage)	8 (50%)	8 (50%)	8 (50%)	9 (56%)
Participants with pets, (percentage)	9 (56%)	11 (69%)	8 (50%)	8 (50%)
Participants using medication*, (percentage)	4 (25%)	5 (31%)	6 (38%)	2 (13%)

*Medication other than antibiotics, proton pump inhibitors, insulin and cancer treatment.

To evaluate the sensitivity of ResCap compared to MSS, we compared the observed number of ARGs identified per sequencing depth, by using rarefaction curves in the same 16 samples per diet group, subjected to both MSS and ResCap. Overall, ResCap was able to detect a higher number of ARGs than MSS. Even at 70 M reads per sample, MSS did not reach the level of sensitivity that ResCap was able to achieve (figure s5a). Where MSS detected 16 ± 5 to 18 ± 4 ARGs per diet group with a sequencing depth of 70 M reads, ResCap resulted in the detection of 33 ± 8 to 39 ± 7 ARGs per diet group with a sequencing depth of 20 M reads (figure s5b).

### Dietary habits are not associated with differences in resistome composition assessed by ResCap

**Figure 4:**
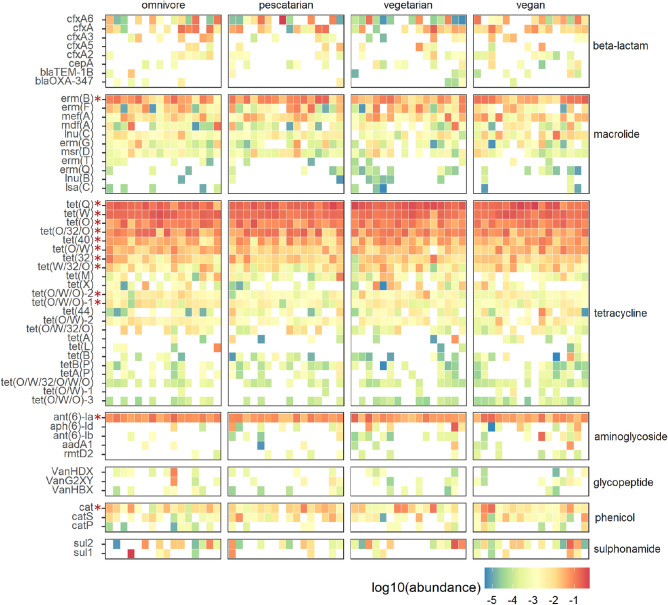
Antibiotic resistance gene distribution in the gut resistome as detected by ResCap. Each column denotes a participant, clustered in columns according to diet group. Rows are categorized by antibiotic resistance gene (ARG) classes. ARGs present at least in 10% of the participants are shown. ARGs that are present in the resistome of at least 95% of the participants are indicated by the asterisks.

A total of 145 ARGs were detected using ResCap, from which 86 ARGs were also detected by MSS (table s1). The majority of genes detected by both methods included tetracycline resistance genes (19 genes, 22%), followed by beta-lactam resistance (19 genes, 22%) and macrolide resistance (15 genes, 17%). The ARGs detected by ResCap and not MSS include mainly beta-lactam (28/59; 47%), tetracycline (9/59; 15%) and aminoglycoside resistance genes (9/59; 15%). In contrast, MSS revealed 32 genes that went undetected by ResCap, including beta-lactam resistance (10 genes, 31%), nitroimidazole resistance (8 genes, 25%), and tetracycline resistance (4 genes, 13%). Of the 145 genes detected by ResCap, 15 ARGs were detected between all diet groups (detected in 95% of the samples) (Fig. 4). These 15 genes included the five genes (*ant(6)-Ia*, *erm*(B)*, tet*(40)*, tet*(Q) and *tet*(W)) that were also detected in all diet groups by MSS. In addition to these five, the chloramphenicol resistance gene *cat*, the lincomycin resistance gene *lnu*(C), the macrolide resistance gene *mef*(A), and the tetracycline resistance genes *tet*(32), *tet*(O), *tet*(O/32/O), *tet*(O/W), *tet*(O/W/O)-1, *tet*(O/W/O)-2 and *tet*(W/32/O) were detected in all diet groups using ResCap (Fig. 4).

**Figure 5:**
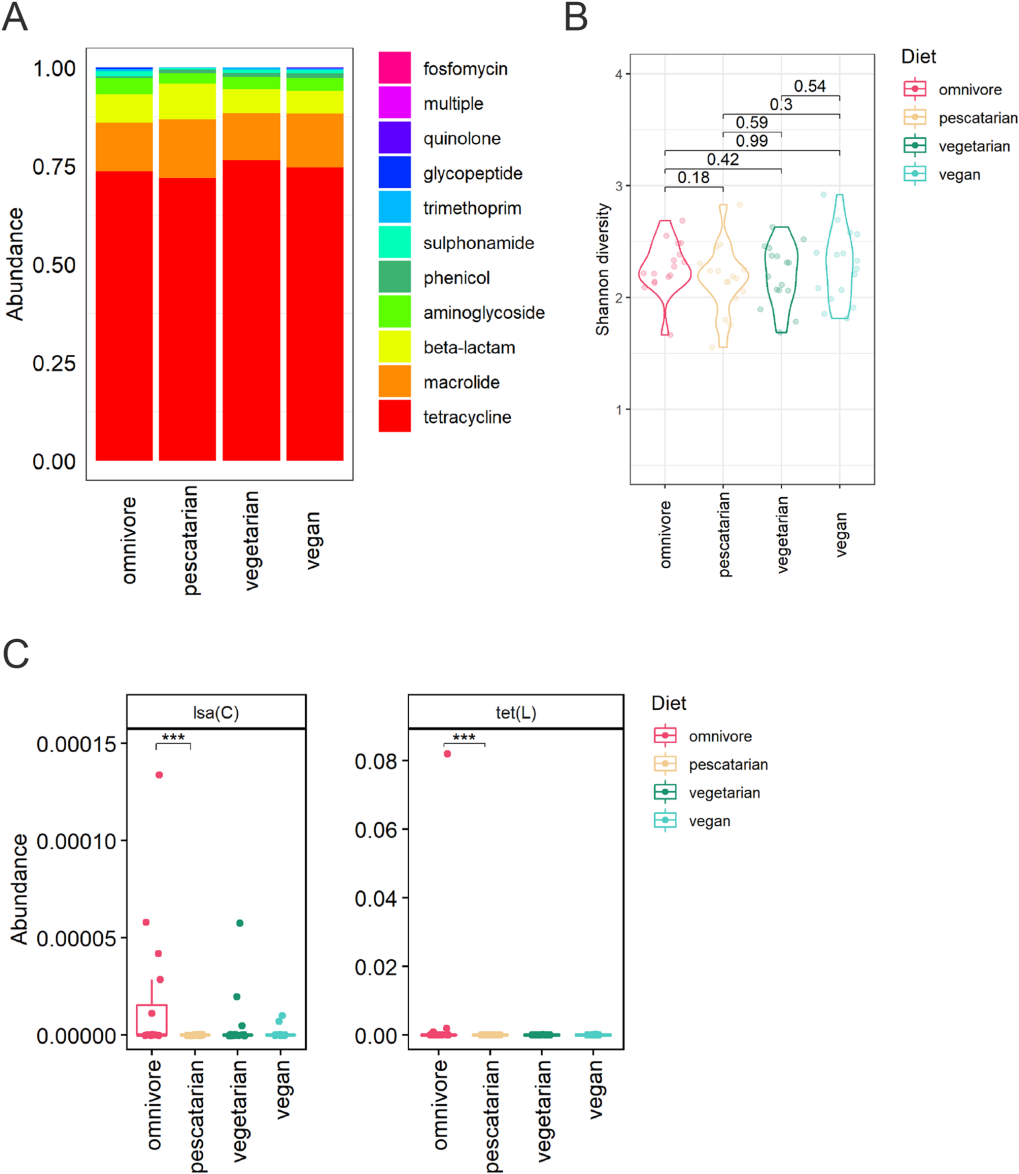
Association of dietary habits and the gut resistome composition through ResCap. (**A**) Relative abundance of the ten most abundant antibiotic resistance gene classes, summarized per diet group. (**B**) Gene diversity per diet group, shown by Shannon index. (**C**) Differential abundance analysis using ANCOM-BC plotted on the scale 0.00–1.00%. Adjusted p values below 0.001 are indicated by ***.

The total number of ARGs detected by ResCap per diet group was not significantly different, with an average of 36 ± 5 genes found in omnivores, 32 ± 9 in pescatarians, 40 ± 10 in vegetarians and 40 ± 10 in vegans (figure s5b). The most abundant ARGs identified encode for tetracycline, macrolide, beta-lactam and aminoglycoside resistance (Fig. 5a). No differences were observed when comparing the overall top 10 most abundant ARG. No significant differences were observed when comparing the resistome diversity as calculated by Shannon index (Fig. 5b). Beta-diversity based PCA further confirmed that diet is not a main driver of the observed variance in resistome composition between the samples (figure s6). Supervised analysis using ANCOM-BC revealed that the abundance of two ARGs, *lsa*(C) and *tet*(L), were significantly different between the diet groups pescatarians and omnivores (Fig. 5c). However, these genes are present in very low abundance (median below 0.05%) and in low prevalence (< 15%). Based on these results we can conclude that differences in dietary habits did not result in significant differences in resistome composition.

## Discussion

By using the combined power of metagenomic shotgun sequencing (MSS) and ResCap we were able to detect over 850 different bacterial species and more than 100 unique antibiotic resistance genes (ARGs) in the gut microbiome of participants from the general Dutch population with distinct dietary habits. Our results show that long-term dietary habits are not associated with specific resistome signatures.

To investigate the gut resistome in humans, we used two techniques, MSS and ResCap. Using genomic DNA from human faecal samples, Lanza et al. found that ResCap results in a two-fold increase in gene diversity compared to MSS. In addition, they reported a 279-fold increase in the amount of reads that mapped to the ResCap targeted genes^46^. Results of our study are comparable to Lanza et al., as we observed a two-fold increase in gene diversity when comparing the number of ARGs detected by ResCap to MSS. Moreover, ResCap resulted in 40.5 ± 15.2% of the reads mapping to ARGs, compared to 0.06 ± 0.03% for MSS, thus indicating a 675-fold change. In a similar way, Macedo et al. also reported an increase of mapped reads of 200-fold, when comparing ResCap to MSS performed on genomic DNA from soil and manure samples^47^. Lastly, Guitor et al. applied a custom ARG probe-database and compared its efficiency to capture the resistome over MSS. They compared both systems using a DNA pool composed of four bacterial species and observed a 100-fold increase of target specific reads^48^. ResCap and MSS sequencing results differed in the number of ARGs that they were able to detect. The 59 ARGs that were detected by ResCap and not MSS suggests that the capture-based approach offers increased sensitivity. However, MSS revealed 32 genes that went undetected by ResCap. From these, 14 genes (44%) were not included in the probe library, including nitroimidazole resistance genes *nimA*, *nimB*, *nimC*, *nimD*, *nimE*, *nimF*, *nimH* and *nimJ* (table s1). We currently have no explanation why we were not able to detect the 14 genes for which probes were present in the ResCap library and that were detected by MSS but not by ResCap.

We observed limited differences in the resistome composition between the diet groups, independently of the detection approach. A previous MSS based comparison of the gut resistome in the Chinese, Danish and Spanish general population, revealed the overall high abundance of ARGs that confer resistance to tetracycline, followed by macrolide, β-lactam and aminoglycoside resistance^26^. This is also observed in our study which included samples from only Dutch population. When using ResCap as high-resolution method to profile the gut resistome, only two ARGs (*lsa*(C) and *tet*(L)) were differentially abundant between omnivores and pescatarians. Since these genes are observed in low abundance and prevalence, the biological relevance of this finding is unclear. MSS revealed that *tet*(X) was more abundant in the resistome of omnivores compared to pescatarians. It is most likely that the increased sample size (n = 149 vs n = 64 samples included for MSS and ResCap respectively) resulted in the detection of differentially abundant *tet*(X) by MSS, but not by ResCap. This suggests that the small sample size for ResCap is a potential limitation of the current study.

Two methods, mOTUs2 and MetaPhlAn3 were used to study the impact of dietary habits on the gut microbiome^49,50^. This showed that long-term dietary habits did not result in significant differences in the top 10 most abundant genera, nor in differences in gut microbiome diversity and that diet was not a main driver of the observed variance in microbiome composition between the samples. Both methods were largely in concordance regarding which genera were differentially abundant using a supervised analysis where only a few compositional differences between the microbiome of omnivores, pescatarians and vegetarians were detected. Variation between methods include the detection of differentially abundant *Clostridium* species by the mOTUs2, compared to differentially abundant species from the family of *Lachnospiraceae*, by the MetaPhlAn3. This variation could be attributed to the distinct ways in which the tools correct for genomic sequences from yet unknown species. While mOTUs2 makes use of marker genes, using its clusters of orthologues groups of proteins approach (COGs), MetaPhlAn3 instead estimates the ‘unknown’ portion by using the average gene length and genome length.

As part of a short-term diet intervention study, David et al. associated the consumption of animal derived products with an increased relative abundance of *Alistipes*, *Bilophila* and *Bacteroides* and a decrease in the relative abundance of *Roseburia*, *Eubacterium rectale* and *Ruminococcus bromii*^14^. Although our study included similar diet groups with omnivores consuming animal derived products, and vegans that do not, we did not observe similar findings. When compared to pescatarians, we detected a lower relative abundance of *Eubacterium* in omnivores, namely *Eubacterium eligens*. Chung et al. described *E. eligens* to be a pectin degrading specialist, which is a major component of plant cell walls and increased plant consumption might therefore explain the lower abundance of *E. eligens* in omnivores^51^. The non-concordance between the studies by David et al. and our study might be explained by differences in resolution of 16S rRNA gene sequencing used in David et al., as compared to MSS in our study, but maybe more importantly this illustrates the difference between an intervention-based study compared to our study design, where long-term diet habits were studied.

More recently, De Angelis et al. compared the gut microbiome, proteome and metabolome of Italian omnivores, vegetarians and vegans based on 16S rRNA gene profiling. Similar to our study, they showed only slight differences in the abundance of bacterial families between the diet groups. *Ruminococcaceae* were found to be most abundant in omnivores, while *Lachnospira* were associated with vegans and vegetarians^52^. Similarly, Losasso et al. observed differences in the relative abundance of *Ruminococcaceae* when comparing the microbiome of vegans, vegetarians, and omnivores^20^, using 16S rRNA profiling. Although the observations are limited to the family level, these studies align with our results, where omnivores were found to have a higher relative abundance of *R. torques* when compared to vegans. In contrast to these dietary studies, we observed differences in the relative abundance of lactic acid bacteria when comparing the microbiome between diet groups. This difference could be explained by the relatively high consumption of dairy products in the Netherlands^53^. Omnivores and pescatarians showed higher relative abundance of *Streptococcus thermophilus* when compared to vegans. Similarly, *Lactococcus lactis* was present in higher relative abundance in the microbiome of pescatarians compared to vegans. Both *S.* *thermophilus* as *L. lactis* have been associated with the consumption of dairy products^54^. Zhernakova et al. also detected a specific association between the consumption of buttermilk and the abundance of *Leuconostoc mesenteroides* and *L. lactis*, when assessing factors contributing to variation of the gut microbiome composition in the Dutch and Belgium population^12,55^. Since dairy products are excluded from the vegan diet, it could explain the low abundance of these lactic acid bacteria in this group.

To conclude, using ResCap we were able to increasingly detect resistance genes in complex samples like human faeces in our study, compared to MSS, and found that the gut microbiome of humans included in this study represented a large reservoir of 145 different antibiotic resistance genes in the studied population. However, differences in long-term dietary habits in the Dutch population did not result in significant differences in resistome composition between the four diet groups. When comparing the microbiome composition of diet groups in the Dutch population, mainly the vegan diet was associated with a distinct taxonomic composition. Since geographic location likely has an impact on the microbiome and resistome composition in the general population, future studies involving human populations from other geographic regions, are needed to determine the generalizability of our findings in the Dutch population.

## Methods

### Participant inclusion

In this study, faecal samples from 149 Dutch individuals from a previous study in the general population (the “NLD-VEGA-study”) were selected based on their dietary habits (Table 1) and categorized in four different diet groups: (1) omnivores, (2) pescatarians, (3) vegetarians and (4) vegans^56^. Groups were matched for sex, age, education level, medication usage and keeping animals (Table 1). Differences between groups were compared by the Kruskal–Wallis or X^2^ test where appropriate. All participants had a Dutch nationality, were born in the Netherlands and lived in urban areas. None of them had used antibiotics, insulin, proton pump inhibitors or drugs related to cancer treatment and chemotherapy in the past 3 months.

### Ethical approval

The original “NLD-VEGA-study” protocol was approved by the medical ethics committee of the University Medical Center Utrecht, the Netherlands (no. 15–561/C). All experiments were performed in accordance with relevant guidelines and regulations.

### Classification of diet groups

Diet groups were defined based on the following criteria: omnivores consumed meat at least three times per week, for the past 6 months or more. Pescatarians consumed fish and animal derived products but did not consume meat in the past 6 months or more. Vegetarians consumed animal derived products but did not consume meat or fish in the past 6 months or more. Vegans did not consume meat, fish, or animal derived products in the past 6 months or more. Furthermore, pescatarians, vegetarians and vegans did not prepare the restricted products described for house members or pets or have house members that consumed those.

### Sample collection, storage and DNA extraction

Faecal samples were sent by regular mail and transported for a maximum of 24 h before storage at − 80 °C. Samples were divided into aliquots of 0.2 g, thereby introducing one freeze–thaw cycle. Samples were thawed a second time and used for DNA extraction, using a modified protocol of the QIAamp fast DNA stool mini kit (Qiagen, Venlo, the Netherlands) as described by Knudsen et al.^57^. In brief, 0.2 g faeces were added to ‘lysing matrix A, 2 ml tubes’ (MP biomedicals, Landsmeer, the Netherlands), containing 1 ml InhibitEx buffer (Qiagen). Beat beating was applied at 3.5 m/s for 30 s, followed by 30 s incubation on ice and one final beat beating step, using the FastPrep-24 (MP biomedicals). After 7 min of incubation at 95 °C, the fast DNA stool mini kit protocol (Qiagen) was resumed at the proteinase K treatment step. Total DNA was quantified by Picogreen assay (Thermo Fisher Scientific, Waltham, MA, USA).

### Metagenomic shotgun sequencing and Microbiome data processing

Samples were sent to Baseclear B.V. (Leiden, the Netherlands) for metagenomic shotgun sequencing (MSS), together with a DNA extraction (negative) control and mock control (ZymoBIOMICS Microbial Community Standard). The NovaSeq 6000 (Illumina, San Diego, USA) was used, with the S1, 2 × 150 bp paired-end kit (Illumina) and the company protocol/standard settings. Raw reads were trimmed by Trimmomatic v0.39 (options: slidingwindow:4:15 minlen:70) and used for taxonomic profiling with either mOTUs2 version 2.5.0 or MetaPhlAn3 using default settings^49,50,58^. Samples contained on average 70.7 M ± 11.3 M reads, while the negative control contained less than 300 k reads. The MSS results of the mock control contained a total of 89 M reads, and matched with the expected mock composition (table s2).

### ResCap sequencing

Sixteen samples per diet group were selected for in-depth resistome analysis using the ResCap targeted sequence capture panel consisting of probes targeting genes that confer resistance to antibiotics, metals, biocides and included probes that target relaxase genes^46^. The capture panel contains probes against 7963 resistance genes and was expanded, by the addition of probes against the following *mcr* genes (*mcr1.1*, *mcr1.2*, *mcr1.3*, *mcr1.4*, *mcr1.5*, *mcr1.6*, *mcr1.7*, *mcr1.8*, *mcr1.9*, *mcr1.10*, *mcr2.1*, *mcr2.2*, *mcr2.3*, *mcr3.1*, *mcr3.2.1*, *mcr3.2.2*, *mcr3.3.1*, *mcr3.3.2*, *mcr3.4.1*, *mcr3.4.2*, *mcr3.5.1*, *mcr3.5.2*, *mcr3.6, mcr4*, *mcr5*, *mcr6*, *mcr7* and *mcr8*) (Roche ID: OID41815). The subset of samples selected per diet group were matched for sex, age, education level, medication usage and having pets (Table 2). Differences between groups were tested by the Kruskal–Wallis or X^2^ test where appropriate. ResCap was performed according to the supplied protocol. In brief, 0.8–1.0 µg DNA was used for fragmentation using the KAPA HyperPlus Kit v4.17 (Roche, Woerden, The Netherlands) to generate 400 bp fragments. End repair, A-tailing and adapter ligation were performed as described by the SeqCap EZ HyperCap User’s Guide v2.3. Pools of 12 samples were used for hybridization and capture using an extended version of the ResCap probe collection as described in the original publication^46^. Sample pools were sequenced on a NextSeq500 (Illumina), using high output and paired-ends of 2 × 150 bp.

### Resistome data processing

ResCap and MSS data were trimmed using Trim Galore version 0.6.4 with standard settings^59^. KMA version 1.3.4 was used to align sequences to the Resfinder database version of 2020-06-02^60^. For KMA, paired-end reads were used as input by using *-ipe*, together with the options: *-tmp, -1t1*, *-cge*, *-apm p*, *-ef*. The resulting list of detected genes and their abundance was trimmed by applying a cut-off of 90% identity (called query Identity) and of 80% coverage (called template coverage). Finally, the output value *depth* was used for subsequent analysis, which represents the amount of aligned base pairs, while correcting for gene length. Analysis of the MSS negative control for antibiotic resistance genes, containing less than 300 k reads, revealed the low abundance of antibiotic resistance genes *tet(L)* and *aadD*.

### Data analysis

Analysis of sequencing data was performed in R version 4.0 and the functions of the packages phyloseq and ggplot2^61–63^. The top 10 abundances of the microbiome and resistome were plotted using aggregate top taxa and plotting functions of the microbiome package^64^. Shannon index was calculated using the alpha diversity functions of the microbiome package and plotting functions of the microbiomeutilities package^65^. Aitchison, Bray–Curtis and Jaccard distance PCA were generated using the transform function of the microbiome package and ordinate (RDA) and plot ordination functions of the phyloseq package. Correlations of sample dissimilarity and diets were tested using PERMANOVA with the adonis function at 999 permutations of the vegan package^66^. Basic accessor functions of phyloseq were used to generate heatmaps. Differential abundance analysis was performed using ANCOM-BC version 1.0.2, with Bonferroni correction for false discovery rate and an alpha of 0.05 as a threshold for significance^45^.

### Consent to publish

In this study, faecal samples from 149 Dutch individuals from a previous study in the population at large (the “vegastudy”) were selected. All participants provided written informed consent for the initial vegastudy and had given permission to use their faecal samples for further research.

## Supplementary Information

Supplementary Information 1.

Supplementary Information 2.

Supplementary Information 3.

## Data Availability

The 149 MSS and 64 ResCap sequencing files have been deposited in the European Nucleotide Archive repository under the study accession no. PRJEB45944 and PRJEB46230, respectively. R scripts to reproduce the analysis reported in this study can be found at; https://gitlab.com/PB_Stege/diet_microbiome_resistome/.

## Abbreviations

ARGs: Antibiotic resistance genes
MSS: Metagenomic shotgun sequencing
PCA: Principal component analysis
ResCap: Resistome capture sequencing approach
