# Supplementary material for: Impact of long-term dietary habits on the human gut resistome in the Dutch population
Source: Sci Rep. 2022 Feb 3;12:1892. doi: 10.1038/s41598-022-05817-4 (PMC8814023; doi:10.1038/s41598-022-05817-4)
Supplement: Supplementary file 1 — Supplementary Information 1. [file 41598_2022_5817_MOESM1_ESM.pdf]

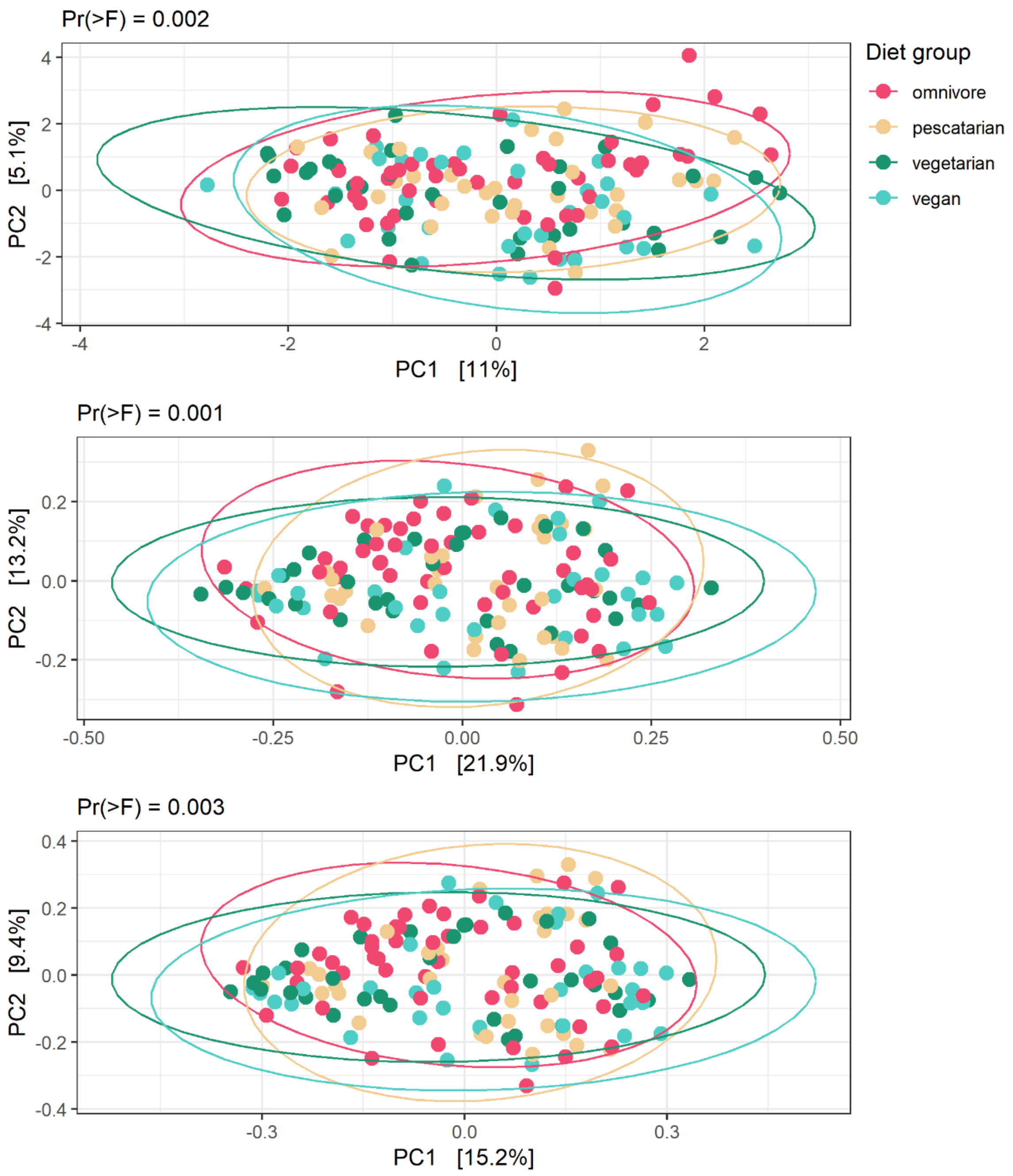

Figure s1 | Overall gut microbiome dissimilarity beta-diversity of all study samples. PCA on bacterial species with data points and ellipses coloured according by diet group, based on (A) Aitchison distance. (B) Bray-curtis distance. (C) Jaccard distance.

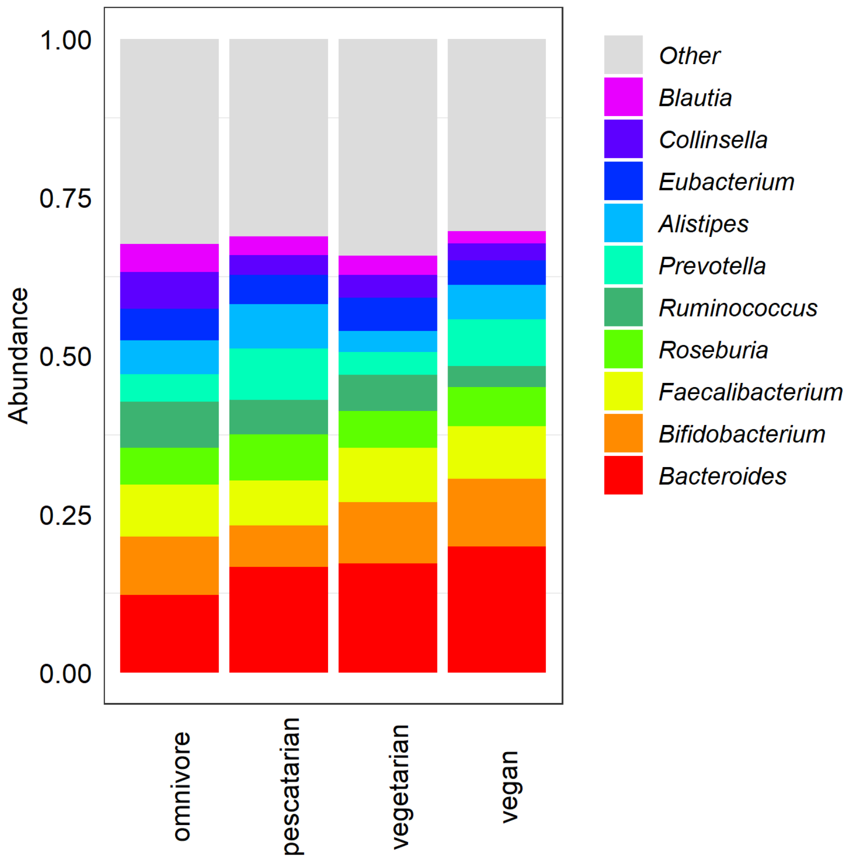

Figure s2 | Association of dietary habits and the gut microbiome composition using MetaPhlAn3. Relative abundance of the 10 most abundant bacterial genera per diet group.

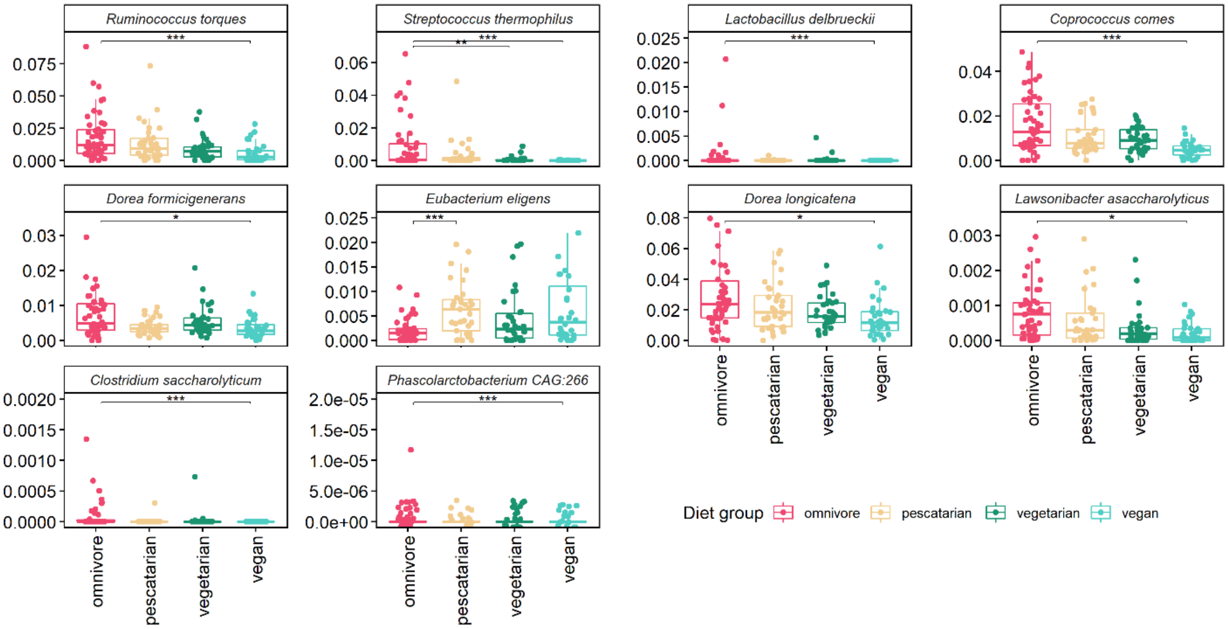

Figure s3 | Differential abundance analysis of gut microbiome composition using MetaPhlAn3 and ANCOM-BC. Plotted on relative abundance scale from 0.00 to 1.00. Adjusted P-values below 0.05 and 0.001 are indicated by * and ***, respectively.

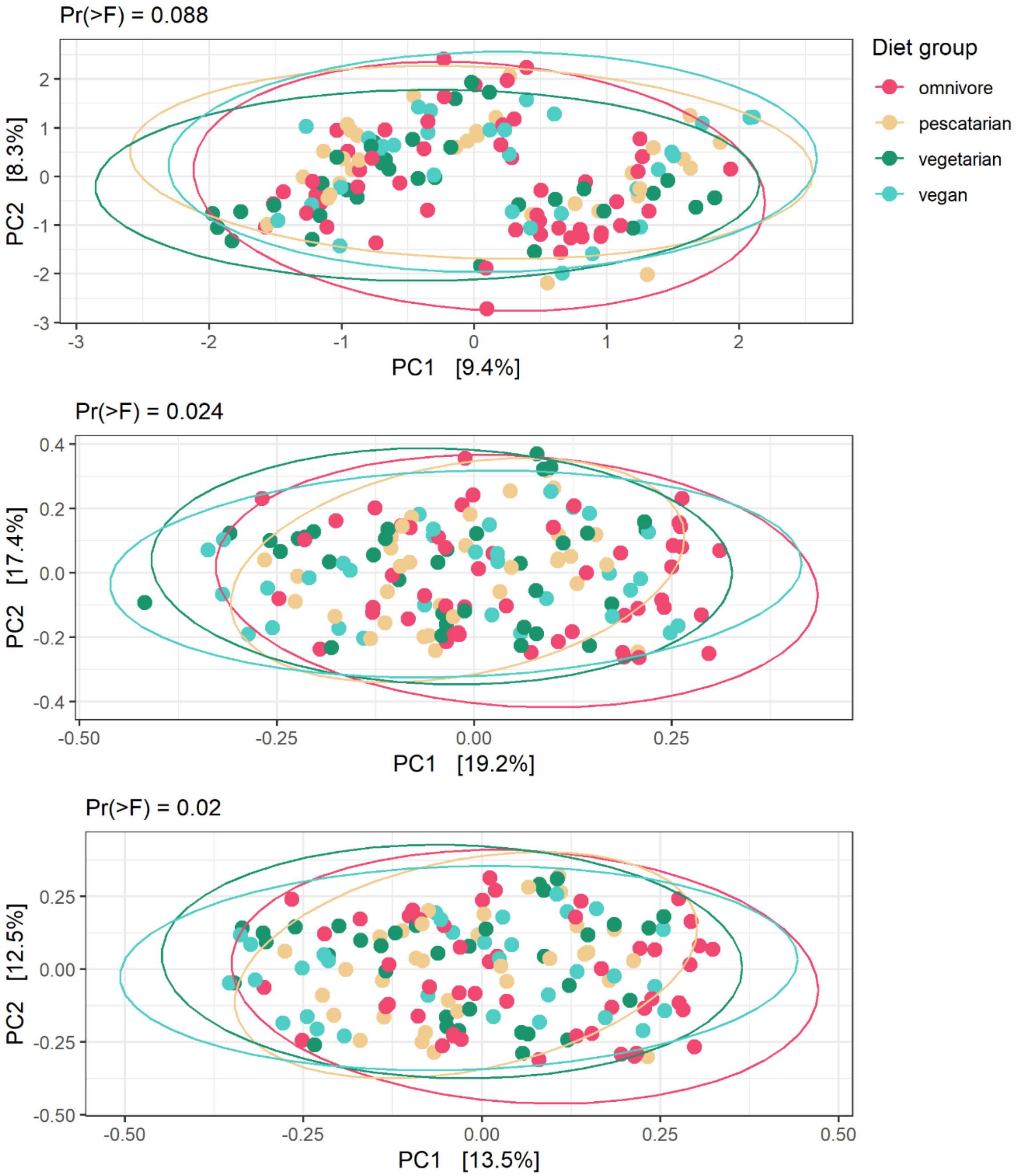

Figure s4 | Overall gut resistome dissimilarity between samples using metagenomic shotgun sequencing. PCA on antibiotic resistance genes with data points and ellipses coloured according by diet group, based on (A) Aitchison distance. (B) Bray-curtis distance. (C) Jaccard distance.

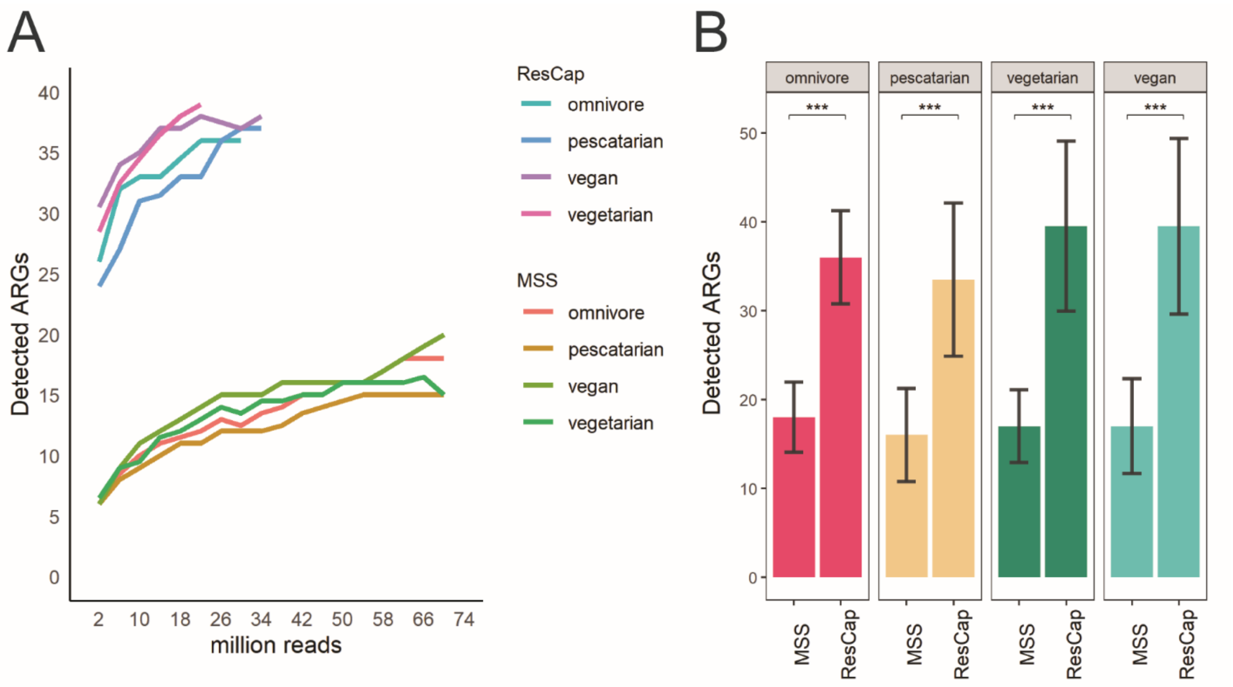

Figure s5 | Comparison of antibiotic resistance genes detected by ResCap and metagenomic shotgun sequencing. (A) Using 64 identical samples, the efficiency to detect antibiotic resistance genes (ARGs) was compared between methods in a rarefaction curve. The number of detected ARGs represents the median of 16 sixteen samples per diet group. Sequencing data was subsampled by steps of four million reads in samples containing up to 70 million reads. (B) The average number of ARGs detected in the 16 samples per diet group, per sequencing methods, with matching standard deviation.

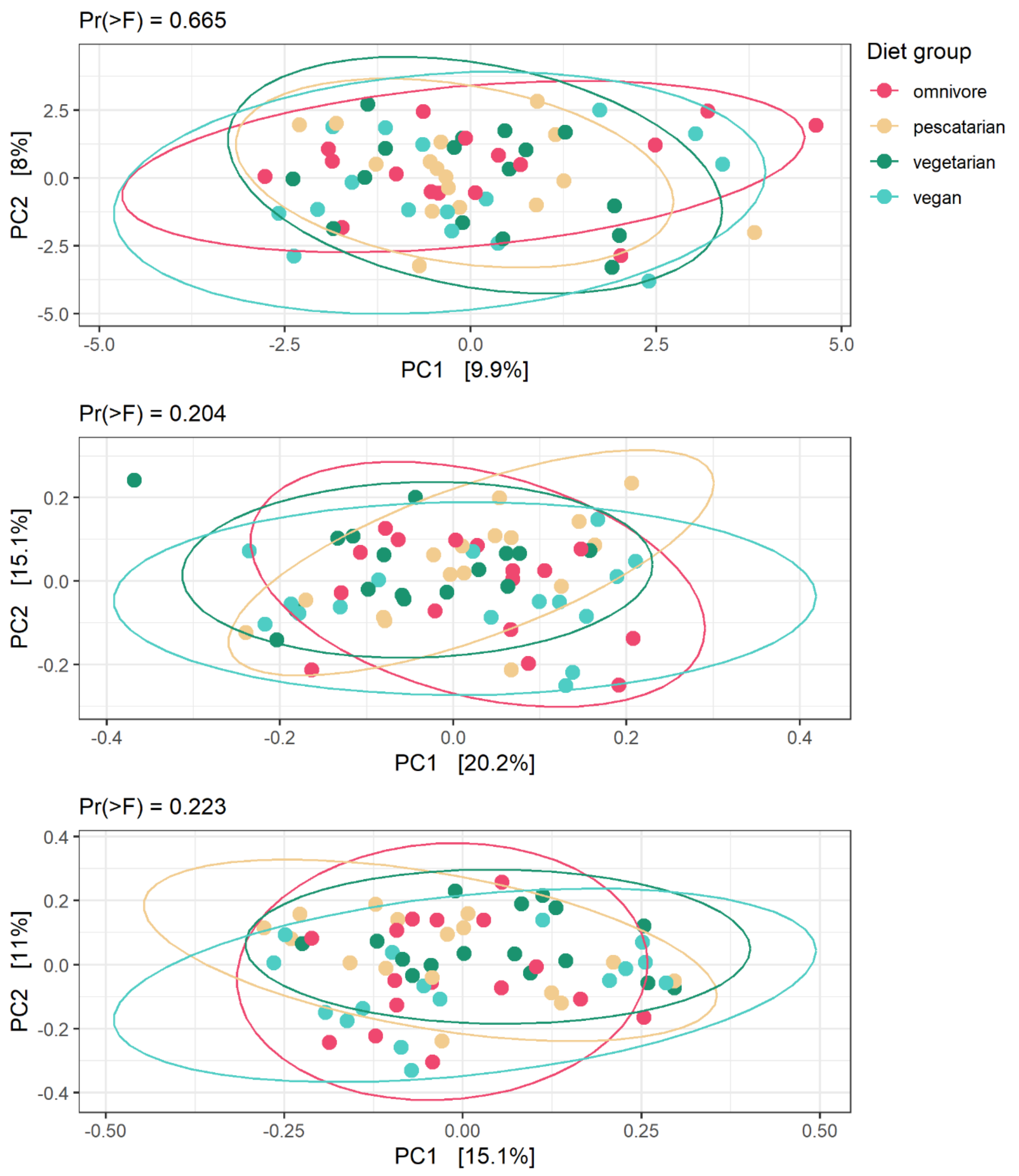

Figure s6 | Overall gut resistome dissimilarity between samples using ResCap. PCA on antibiotic resistance genes with data points and ellipses coloured according by diet group, based on (A) Aitchison distance. (B) Bray-curtis distance. (C) Jaccard distance.
