# Supplementary material for: Impact of long-term dietary habits on the human gut resistome in the Dutch population
Source: Sci Rep. 2022 Feb 3;12:1892. doi: 10.1038/s41598-022-05817-4 (PMC8814023; doi:10.1038/s41598-022-05817-4)
Supplement: Supplementary file 2 — Supplementary Information 2. [file 41598_2022_5817_MOESM2_ESM.pdf]

Table S1. List of all antibiotic resistance genes detected by both techniques (shotgun metagenome sequecing and Rescap) or only one of them. Hilighted in grey are the genes that do not have a probe for capture in the Rescap technique.

Antibiotic resistance genes detected by		
**ResCap and MSS**	**only ResCap**	**only MSS**
aac(3)-IId	aac(3)-IIa	aac(3)-IV
aadA1	aac(3)-IIc	aadD
aadA2	aac(3)-IIe	aph(4)-Ia
aadA5	aadA15	blaCMY-150
ant(6)-Ia	aadA4	blaGES-1
ant(6)-Ib	aadA8b	blaMIR-2
aph(6)-Id	blaACT-10	blaOXA-232
blaACI-1	blaACT-14	blaOXY-2-6
blaACT-12	blaACT-16	blaSHV-185
blaCMY-48	blaACT-4	blaTEM-1C
blaCMY-82	blaACT-5	cfiA1
blaCTX-M-15	blaACT-6	cfiA2
blaCTX-M-32	blaACT-7	cfr(C)
blaOXA-347	blaACT-9	crpP
blaOXY-2-7	blaCMY-110	ere(D)
blaTEM-116	blaCMY-117	erm(C)
blaTEM-1B	blaCMY-51	lnu(P)
cat	blaCTX-M-101	mecA
catA1	blaLEN21	nimA
catP	blaMIR-5	nimB
catQ	blaOKP-B-5	nimC
catS	blaOXA-1	nimD
cepA	blaOXA-212	nimE
cepA-29	blaOXA-395	nimF
cepA-44	blaOXA-486	nimH
cepA-49	blaOXY-2-2	nimJ
cfr(B)	blaPAO	qnrB19
cfxA	blaPLA1a	qnrB38
cfxA3	blaSHV-48	tet(D)

Absent in the ResCap probe database

cfxA4	blaTEM-102	tet(S)
cfxA5	blaTEM-182	tetA(46)
cfxA6	blaTEM-1A	tetB(46)
dfrA1	blaTEM-40	
dfrA12	cat(pC194)	
dfrA14	catB7	
dfrA17	cfxA2	
dfrA7	cmr	
erm(B)	erm(A)	
erm(F)	fosA5	
erm(G)	lnu(A)	
erm(Q)	lnu(B)	
erm(T)	mre(A)	
erm(X)	npmA	
floR	qnrS5	
fosA	rmtD	
fosA2	rmtG	
lnu(C)	tet(39)	
lsa(A)	tet(O/W/32/O)	
lsa(C)	tet(O/W/32/O/W/O)	
lsa(E)	tet(O/W/O)-1	
mdf(A)	tet(O/W/O)-3	
mef(A)	tet(S/M)	
mph(A)	tet(W/32/O)	
msr(C)	tet(X4)	
msr(D)	VanC2XY	
oqxA	VanC3XY	
oqxB	VanC4XY	
qepA4	VanGXY	
qnrS1	VanXY	
rmtD2		
rmtF		
sul1		
sul2		

tet(32)
tet(40)
tet(44)
tet(A)
tet(B)
tet(L)
tet(M)
tet(O)
tet(O/32/O)
tet(O/W)
tet(O/W)-1
tet(O/W)-2
tet(O/W/O)-2
tet(Q)
tet(W)
tet(X)
tet(X6)
tetA(P)
tetB(P)
VanG2XY
VanHBX
VanHDX
vat(E)
