# Supplementary material for: Impact of long-term dietary habits on the human gut resistome in the Dutch population
Source: Sci Rep. 2022 Feb 3;12:1892. doi: 10.1038/s41598-022-05817-4 (PMC8814023; doi:10.1038/s41598-022-05817-4)
Supplement: Supplementary file 3 — Supplementary Information 3. [file 41598_2022_5817_MOESM3_ESM.pdf]

Table S2. Description of the mock community composition used as positive control in the shotgun metagenome analysis. Relative abundance is shown as determined by the manufracture, when analyzed with mOTUs2 or Metaphlan 3.

Expected mock commnunity composition

**species**	**relative abundance (%)**
Salmonella enterica	12
Enterococcus faecalis	12
Escherichia coli	12
Pseudomonas aeruginosa	12
Staphylococcus aureus	12
Lactobacillus fermentum	12
Bacillus subtilis	12
Listeria monocytogenes	12
Saccharomyces cerevisiae	2
Cryptococcus neoformans	2

ZymoBIOMICS Microbial Community Standard

Mock community composition (mOTUs2)

**detected species**	**relative abundance (%)**
s__Salmonella enterica/bongori	18
s__Bacteria s.	18
s__Proteobacteria sp.	17
s__Pseudomonas	11
s__Staphylococcus aureus	11
s__Lactobacillus fermentum	9
s__Bacteria sp.	8
s__Listeria monocytogenes	7
other	1

Mock community composition (Metaphlan3)

**detected species**	**relative abundance (%)**
s__Salmonella_enterica	20
s__Enterococcus_faecalis	18
s__Escherichia_coli	21
s__Pseudomonas_aeruginosa_gr	4
s__Staphylococcus_aureus	12
s__Lactobacillus_fermentum	10
s__Bacillus_intestinalis	7
s__Listeria_monocytogenes	6
other	1
